# Association of Common Variants of *APOE*, *CETP*, and the 9p21.3 Chromosomal Region with the Risk of Myocardial Infarction: A Prospective Study

**DOI:** 10.3390/ijms241310908

**Published:** 2023-06-30

**Authors:** Sergey Semaev, Elena Shakhtshneider, Liliya Shcherbakova, Pavel Orlov, Dinara Ivanoshchuk, Sofia Malyutina, Valery Gafarov, Mikhail Voevoda, Yuliya Ragino

**Affiliations:** 1 Institute of Cytology and Genetics, Siberian Branch of Russian Academy of Sciences (ICG SB RAS), 10 Prospekt Ak. Lavrentyeva, Novosibirsk 630090, Russia; 2 Institute of Internal and Preventive Medicine (IIPM)-Branch of ICG SB RAS, 175/1 Borisa Bogatkova Str., Novosibirsk 630089, Russia

**Keywords:** rs1333049, rs708272, rs7412, rs429358, myocardial infarction, cardiovascular disease, prospective study

## Abstract

The individual risk of an unfavorable cardiovascular outcome is determined by genetic factors in addition to lifestyle factors. This study was aimed at analyzing possible associations of several genetic factors with the risk of myocardial infarction (MI). For our study, we selected genes that have been significantly associated with MI in meta-analyses: the chromosomal region 9p21.3, the *CETP* gene, and the *APOE* gene. In total, 2286 randomly selected patients were included. Rs708272 and rs429358 and rs7412 were analyzed using RT-PCR via the TaqMan principle, and rs1333049 vas analyzed via a commercial KASP assay. In our sample, the frequencies of alleles and genotypes were consistent with frequencies in comparable populations of Eastern and Western Europe. Allele C of rs1333049 was significantly associated with MI among males (*p* = 0.027) and in the whole study sample (*p* = 0.008). We also revealed a significant association of the ɛ2/ɛ4 genotype of *APOE* with MI among males (*p* < 0.0001) and in the whole study sample (*p* < 0.0001). Thus, among the tested polymorphisms, some genotypes of rs1333049 and rs429358 and rs7412 are the most strongly associated with MI and can be recommended for inclusion into a genetic risk score.

## 1. Introduction

Cardiovascular diseases (CVDs) are the leading cause of death in industrialized countries [1]. An important and relevant task for the healthcare system is to identify the groups most susceptible to CVDs. The individual risk of an unfavorable cardiovascular outcome is determined by genetic factors in addition to conventional risk factors (smoking, arterial hypertension, dyslipidemia, diabetes mellitus, and abdominal obesity) and unconventional ones, including psychological risk factors (stress, anxiety and depression, income level, marital status, and domestic conflicts). The relative risk of new coronary events in patients with high genetic risk is 90% higher than that in people with low genetic risk [2]. It has been shown that structural changes in DNA independently affect the overall mortality caused by cardiovascular events and myocardial infarction (MI) [2,3,4,5]. The risk of an adverse outcome depends on the presence of a certain allele or genotype.

For our study, we selected genes that have been significantly associated with MI or coronary artery disease (CAD) in meta-analyses: chromosomal region 9p21.3 [6,7,8], the *CETP* gene [9,10], and the *APOE* gene [11,12].

In a meta-analysis of the rs1333049 SNP in 12,004 cases and 28,949 controls, H. Schunkert et al. revealed an increase in the overall level of evidence for an association with CAD to *p* = 6.04 × 10^−10^ (odds ratio [OR] 1.24, 95% confidence interval [CI] 1.20–1.29). The genotyping of 31 additional SNPs in the region identified several with a highly significant association with CAD, but none had predictive information beyond that of rs1333049 [6]. C.J. O’Donnel et al. conducted a meta-analysis and revealed that SNPs in 9p21 strongly correlate with coronary artery calcification and MI [7]. The 9p21.3 chromosomal region contains two genes (encoding proteins CDKN2A and CDKN2B) and the gene of long noncoding RNA ANRIL (in the antisense orientation at the *INK4* locus) [13].

There is a correlation between the level of high-density lipoprotein cholesterol (HDL-C) and the risk of CVD; this correlation is in part explainable by the participation of HDL in reverse cholesterol transport [14]. The *CETP* gene is located on the 16th chromosome (16q21) [5]. Cholesterol ester transporter CETP is a hydrophobic glycoprotein involved in the transfer of esterified cholesterol from HDLs to very low-density lipoproteins and intermediate-density lipoproteins, with the conversion of the latter into low-density lipoproteins (LDLs) [5]. Rs708272 of the *CETP* gene is associated with a high risk of coronary heart disease and the progression of coronary atherosclerosis, and is a predictor of the response to statin therapy [5]. Q. Wang et al. performed a meta-analysis to evaluate the relations of seven functional polymorphisms in the *CETP* gene with the risk of MI and found that polymorphisms rs708272 (C>T) and rs1800775 (C>A) may contribute to MI susceptibility, especially among white populations; Q. Wang et al. have hypothesized that rs708272 and rs1800775 may be promising potential biomarkers for early diagnosis of MI [9]. M. Cao et al. have conducted a meta-analysis (13 studies involving 8733 MI cases and 8573 controls), and the results suggest that the B2B2 genotype of the *CETP* TaqIB polymorphism is a protective factor against the development of MI [10].

Apolipoprotein E (APOE) is a major chylomicron apolipoprotein and is required for the normal catabolism of triglyceride-rich lipoprotein components [15,16]. The results of a recent meta-analysis conducted by A. Shao et al. revealed that *APOE* ɛ2-involving genotypes may be protective factors against MI; in contrast, ɛ4-involving genotypes may be risk factors for MI [11]. These results are consistent with findings of a meta-analysis of *APOE* gene polymorphism and susceptibility to MI performed by H. Xu et al. in 2014 [12].

Taking into account the meta-analyses of the associations of genes *APOE* and *CETP* and chromosomal region 9p21.3 with the risk of MI [6,7,8,9,10,11,12], data from pilot studies showing an association of these variants with the risk of MI in other populations [4,5], and preliminary data on the correlation of these variants with metabolic disorders leading to MI [6,14,15,16], we chose common variants in *APOE*, *CETP*, and genes located in 9p21.3 to evaluate their possible association with MI.

Our aim was to examine a possible association of rs1333049, rs708272, and rs7412 and rs429358 with the risk of MI in a prospective study.

## 2. Results

### 2.1. The Main Characteristics of the Study Sample

The main characteristics of the study population are presented in Table 1. The ratio of males to females was 0.43:0.57.

Females had higher levels of TC, HDL-C, and LDL-C and a higher body mass index, while the atherogenic coefficient and the level of triglycerides did not differ statistically significantly from those in males.

### 2.2. Frequencies of Alleles and Genotypes of rs1333049, rs708272, and rs429358 and rs7412 (the APOE Gene)

The frequencies of rs1333049 alleles in the study sample are listed in Table 2. The frequency of allele C of rs1333049 was found to be consistent with its frequency in comparable populations of Eastern and Western Europe according to gnomAD Genomes European data: C frequency = 0.4858, G frequency = 0.5142 [17].

The frequency of allele A of *CETP* rs708272 in our sample (Table 3) proved to be consistent with its frequency in comparable populations of Eastern and Western Europe according to gnomAD Genomes European data: G frequency = 0.5772, A frequency = 0.4278 [18].

It was found that for rs429358 and rs7412 in the study sample (*n* = 2286), the distribution of genotype frequencies conforms to the Hardy–Weinberg equilibrium (χ^2^ = 0.94 and χ^2^ = 0.03, respectively). The frequency of a minor allele (C) of rs429358 is 0.13, and the frequency of a minor allele (T) of rs7412 is 0.09 in the study sample from Western Siberia, consistent with the distribution of allele frequencies in comparable populations of Eastern and Western Europe; according to gnomAD Genomes European, for rs429358 C the frequency is 0.1486 [19], and for rs7412 T the frequency is 0.0767 [20].

In our study, the ε3 allele turned out to be the most common: frequency of 0.7829 in the male subgroup, 0.7854 in the female subgroup, and 0.7843 for the total study population (Table 4).

### 2.3. Association of Studied Variants with MI

During a 10-year period (2005–2015), in the main representative sample in another study (*n* = 9360), the present investigators collected data on new cases of MI at the Myocardial Infarction Registry of Novosibirsk City [21]. During this observation period, 509 new cases of MI were registered. In the current study sample (*n* = 2286), which was genotyped for rs1333049, rs708272, and rs7412 and rs429358, there were 183 new cases of MI during the 2005–2015 period.

In the sample from Western Siberia, we confirmed the association of rs1333049 with MI among males (*p* = 0.027) and in the whole study population (*p* = 0.008) (Table 5). Therefore, carriage of the C allele is a risk factor of MI.

We found no significant association of rs708272 with MI in our sample (Table 6).

We confirmed a significant association of the ɛ2/ɛ4 genotype of the *APOE* gene with the risk of MI among males (*p* < 0.0001) and in the whole study population (*p* < 0.0001) (Table 7).

## 3. Discussion

In our study, carriage of the C allele of rs1333049 was found to be a risk factor for MI in a population sample from Western Siberia. The minor allele (risk allele) of rs1333049 (C) is widespread across the globe. This allele raises the risk of CAD by 15–20% in the heterozygous state and by 30–40% in the homozygous state [22,23]. According to the literature, chromosomal locus 9.21, where rs1333049 is located, may be involved in the signaling pathway related to inflammation in the arterial wall [24] and to coronary artery calcification, which underlies most cases of MI [7]. The association of rs1333049 with CAD and MI has been found in various ethnic groups in Russia and elsewhere [25,26,27,28,29].

High plasma levels of CETP are correlated with low HDL-C levels and have been shown to be a strong risk factor for CVD, including MI [9]. Although MI is one of the most common heritable CVDs, the underlying molecular pathways remain undefined [9]. For instance, it has been speculated that CETP genetic variants may be involved in the development of MI. In a meta-analysis, Wang Qi et al. revealed that *CETP* polymorphism rs708272 (C/T) might increase the risk of MI, especially among white populations, whereas such a relation was not observed among Asian populations [9]. In one of our previous studies, we found a significant association of rs708272 of the *CEPT* gene with fatal cases of MI [5]. In the present work, we did not find a significant correlation of rs708272 with MI. 

Associations of *APOE* polymorphism and MI risks have been investigated extensively [11]. In 2014, H. Xu et al. performed a meta-analysis, finding that the frequency of MI increases for ε4ε4 vs. ε3ε3 (OR 1.59, 95% CI 1.15–2.19, *p* = 0.005); conversely, no significant association was detected for ε2ε2 vs. ε3ε3 (OR 0.73, 95% CI 0.40–1.32, *p* = 0.29) [12]. In contrast, a meta-analysis published in 2015 revealed that for ε2ε2 vs. ε3ε3 the frequency of MI decreased (OR 0.40, 95% CI 0.20–0.83, *p* = 0.00), except in white and Asian populations, and no significant association existed for ε4ε4 vs. ε3ε3 (OR 1.34, 95% CI 0.91–1.98, *p* = 0.186) in these populations [11]. The *APOE* ε4 allele is considered one of the most notorious common genetic risk factors, with an adverse effect on lipid profiles and CVDs, whereas the rare allele ε2 is often regarded as a protective rare variant [16]. On the other hand, A. Lumsden et al. obtained evidence that the ε2 allele, which is typically considered beneficial, raises the risk of several conditions when homozygous [16]. In the present paper, we revealed a significant association of the ε2ε4 genotype with MI among males (*p* < 0.0001) and in the whole study population (*p* < 0.0001), in agreement with the results of the meta-analysis performed by H. Xu et al. [12]. In contrast, we detected no association of genotypes ε3ε4 and ε4ε4 with a higher risk of MI. In our study, ε3 allele frequencies were consistent with data on the frequency of this allele worldwide [16,30,31]. Frequencies of alleles ε2 and ε4 were comparable with the distribution of frequencies in comparable populations of Eastern and Western Europe [32,33]. 

Our study has some limitations. We analyzed only rs1333049 (which is located in the 9p21.3 region), rs708272 of the *CEPT* gene, and rs7412 and rs429358 of the *APOE* gene and thus could not rule out the influence of other factors that may affect the results of observational studies. Our sample included participants of mostly European ancestry (>90%). The lack of ethnic diversity in genetic studies conducted to date is widely documented, with most originating from white, Western, and European ancestry groups. For example, in the first 10 years of genetic risk score research, 67% of studies included exclusively European-ancestry participants, and 19% only East-Asian-ancestry participants. Only 3.8% of articles from this time period included cohorts of African, Hispanic, or indigenous peoples, highlighting huge disparities in genetic research populations [34]. The complexity of investigating the role of genetic factors lies in the fact that a study conducted in one population cannot be applied to another population without taking into account population structure [35,36]. 

The level of individual risk of a long-term unfavorable outcome of CVDs is due to both genetic factors and lifestyle factors. Within the framework of this work, the results of a study are presented in which data were obtained on a number of genetic factors, contributing to an increase in the risk of an unfavorable outcome of CVD. Structural changes in DNA independently affect the overall mortality from cardiovascular events and MI, consistent with the results of previous studies [5]. The risk of an adverse outcome varies depending on the presence of a certain allele or genotype. Investigation into genetic risk factors of a long-term adverse outcome of CVD is important not only for the analysis of the outcomes of the disease but also for prevention, given that it is possible to determine genetic variations before the first clinical manifestations of the disease. Patients with high genetic risk may receive additional motivation to adhere to a healthy lifestyle.

It is advisable to expand the research on genetic risk factors of poor long-term CVD outcomes both by increasing the number of validated genetic variants and by verifying the results obtained in genetically diverse populations of various ethnic groups. Furthermore, the best predictive models may be constructed from genetic risk scores based on a large number of SNPs that have not necessarily reached genome-wide or even statistical significance separately [37]. In conclusion, among the polymorphisms evaluated in the present study, some genotypes of rs1333049 (chromosomal region 9p21.3) and of rs429358 and rs7412 (the *APOE* gene) proved to be most closely associated with MI and can be recommended for inclusion in a genetic risk score.

## 4. Materials and Methods

### 4.1. The Study Sample

A cross-sectional epidemiological examination of adult inhabitants was carried out in Novosibirsk (Western Siberia, Russia). The study involved materials from the “Collection of human biomaterials at the Institute of Internal and Preventive Medicine—a branch of ICG SB RAS” (No. 0324-2017-0048). The profile of the group of residents in the surveyed districts was typical for the city of Novosibirsk in terms of ethnicity, age, and employment status [38]. From Novosibirsk residents examined within the framework of an international multicenter study on risk factors of CVDs in Eastern Europe (HAPIEE; Health, Alcohol, and Psychosocial Factors in Eastern Europe) [38], using a random-number table, a representative sample was chosen previously (9360 subjects, 45–69 years old, age 53.8 ± 7.0 years [mean ± SD], males/females ratio 50/50, white ethnicity > 90%). The study protocol was approved by the ethics committee at the Institute of Internal and Preventive Medicine—a branch of the Institute of Cytology and Genetics (ICG), the Siberian Branch of the Russian Academy of Sciences (SB RAS), Novosibirsk, Russia. From each patient, we obtained informed consent to be examined for the collection and analysis of biological samples.

### 4.2. Measures and Clinical Data

The program of clinical examination included the registration of sociodemographic data; a standard questionnaire on smoking and alcohol use; a history of chronic diseases; the use of medications; the Rose cardiological questionnaire; anthropometric data (height, body weight, and waist circumference); three-time measurement of blood pressure; spirometry; electrocardiography; detection of “definite coronary heart disease” in accordance with validated epidemiological criteria (MI as determined by electrocardiography, pain-free coronary heart disease according to electrocardiography, or stable effort angina of functional classes II–IV according to the Rose questionnaire) and clinical-functional criteria (according to electrocardiograms interpreted via the Minnesota code); and biochemical assays of blood serum (total cholesterol, HDL-C, triglycerides, and fasting glucose). Blood sampling from the cubital vein was performed in the morning on an empty stomach and at 12 h after a meal. Blood lipid profiling (total cholesterol, triglycerides, HDL-C, and LDL-C) was conducted via enzymatic methods using standard reagents (Biocon Fluitest; Lichtenfels, Germany) on a Labsystem FP-901 biochemical analyzer (Helsinki, Finland). The atherogenic coefficient was calculated using the formula: IA = (TC − HDL-C)/HDL-C.

Data collection in the cohort regarding endpoints (MI) was performed from several sources of information: (i) the second clinical examination of the same group in 2013–2015, and (ii) a database called the Myocardial Infarction Registry of Novosibirsk City. The MI group consisted of 183 people (128 males, 55 females). Inclusion criteria were as follows: MI that occurred during the observation period (according to all registries); MI in the anamnesis as confirmed by instrumental examination methods. An exclusion criterion was a history of MI not confirmed by instrumental examination methods.

### 4.3. Genotyping and Quality Control

For molecular genetic research, 2690 subjects were selected from the aforementioned main sample using the random-number method. After excluding unconfirmed cases of MI and non-MI deaths, the sample size was 2286. Phenol–chloroform extraction was carried out to isolate DNA from the blood samples [39]. The quality of the extracted DNA was assessed using an Agilent 2100 Bioanalyzer capillary electrophoresis system (Agilent Technologies Inc., Santa Clara, CA, USA).

Rs1333049 was genotyped using the commercial KASP assay [40] designed by Biolabmix (BioLabMix, Novosibirsk, Russia) and the HS-qPCR Hi-ROX (2×) (BioLabMix, Novosibirsk, Russia) on a StepOnePlus Real-Time PCR System (Thermo Fisher Scientific, Foster City, CA, USA).

Genotyping of rs708272 was conducted by means of TaqMan single-nucleotide polymorphism (SNP) Genotyping Assays (Thermo Fisher Scientific, Foster City, CA, USA) and the BioMaster HS-qPCR HI-ROX Kit (Biolabmix, Novosibirsk, Russia) on the StepOnePlus Real-Time PCR System (Thermo Fisher Scientific, Foster City, CA, USA).

Genotyping of rs429358 and rs7412 was performed with allele-specific real-time PCR with fluorescence detection according to the TaqMan principle (Biolink, Novosibirsk, Russia) on the StepOnePlus Real-Time PCR System (Thermo Fisher Scientific, Foster City, CA, USA). 

Laboratory personnel performing the genotyping assays were blinded to the physical and clinical-examination data.

### 4.4. Statistical Analyses

The analyses of the data were carried out using the statistical software package SPSS for Windows. The significance of differences in allele frequencies among the studied groups and conformance to the Hardy–Weinberg equilibrium were evaluated with the χ^2^ test. The strength of association between the investigated variants and MI among males, females, and in the whole study sample was assessed by means of ORs with the corresponding 95% CI.

## Figures and Tables

**Table 1:** Baseline characteristics of the study population (*n* = 2286) randomly selected for molecular genetic analysis.

	Males	Females	Total
Number of subjects, *n*	981	1305	2286
Age, years	58.0 ± 6.9	58.0 ± 7.2	58.0 ± 7.1
TC, mg/dL	239.7 ± 49.7	259.4 ± 58.1 **	250.9 ± 55.5
HDL-C, mg/dL	58.2 ± 15.0	61.2 ± 14.2 **	59.9 ± 14.6
LDL-C, mg/dL	153.3 ± 43.9	169.3 ± 51.2 **	162.4 ± 48.9
TGs, mg/dL	140.9 ± 80.6	142.9 ± 82.7	142.0 ± 81.8
Atherogenic coefficient	3.3 ± 1.3	3.4 ± 1.5	3.4 ± 1.4
Body mass index, kg/m^2^	27.2 ± 4.7	30.1 ± 5.4 **	28.8 ± 5.3

Continuous variables are presented as mean ± standard deviation. HDL-C, high-density lipoprotein cholesterol; LDL-C, low-density lipoprotein cholesterol; TC, total cholesterol; TGs, triglycerides. ** *p* < 0.0001 for the comparison of males with females.

**Table 2:** Frequencies of alleles and genotypes of rs1333049 (located in the 9p21.3 region).

	Males (*n* = 972)	Females (*n* = 1300)	Total (*n* = 2272)
	%	%	%
**Genotypes**			
CC	20.9 *n* = 203	22.7 *n* = 295	21.9 *n* = 498
CG	53.3 *n* = 518	48.0 *n* = 624	50.3 *n* = 1142
GG	25.8 *n* = 251	29.3 *n* = 381	27.8 *n* = 632
**Alleles**			
C	47.5	46.7	47.1
G	52.5	53.3	52.9

*n*: the number of individuals.

**Table 3:** Frequencies of alleles and genotypes of rs708272 of the *CEPT* gene.

	Males (*n* = 981)	Females (*n* = 1305)	Total (*n* = 2286)
	%	%	%
**Genotypes**			
AA	22.7 *n* = 223	20.6 *n* = 269	21.5 *n* = 492
AG	44.5 *n* = 436	50.5 *n* = 659	47.9 *n* = 1095
GG	32.8 *n* = 322	28.9 *n* = 377	30.6 *n* = 699
**Alleles**			
A	44.9	45.9	45.5
G	55.1	54.1	54.5

*n*: the number of individuals.

**Table 4:** Frequencies of alleles and genotypes of rs429358 and rs7412 of the *APOE* gene.

	Males (*n* = 981)	Females (*n* = 1305)	Total (*n* = 2286)
	%	%	%
**Genotypes**			
ɛ2/ɛ4	2.9 *n* = 28	2.1 *n* = 27	2.4 *n* = 55
ɛ2/ɛ2	0.7 *n* = 7	0.8 *n* = 11	0.8 *n* = 18
ɛ2/ɛ3	14.9 *n* = 146	12.3 *n* = 161	13.4 *n* = 307
ɛ3/ɛ3	61.8 *n* = 606	62.1 *n* = 810	61.9 *n* = 1416
ɛ3/ɛ4	18.1 *n* = 178	20.6 *n* = 269	19.6 *n* = 447
ɛ4/ɛ4	1.6 *n* = 16	2.1 *n* = 27	1.9 *n* = 43
**Allele frequencies**			
ε2	9.6	8.1	8.7
ε3	78.3	78.5	78.4
ε4	12.1	13.4	12.9

*n*: the number of individuals.

**Table 5:** Associations of rs1333049 genotypes with MI.

Sex	Genotype	Study Sample		Myocardial Infarction		OR (95% CI)	*p*
		*n*	%	*n*	%		
Males	CC	167	19.8	36	28.3	1.606 (1.054–2.448)	0.027 *
	CG	459	54.3	59	46.5	0.730 (0.502–1.061)	0.098
	GG	219	25.9	32	25.2	0.963 (0.626–1.479)	0.863
Females	CC	277	22.2	18	32.7	1.700 (0.953–3.003)	0.069
	CG	601	48.3	23	41.8	0.770 (0.446–1.331)	0.348
	GG	367	29.5	14	25.5	0.817 (0.440–1.517)	0.521
Total	CC	444	21.2	54	29.7	1.564 (1.119–2.186)	0.008 *
	CG	1060	50.7	82	45.0	0.797 (0.588–1.080)	0.143
	GG	586	28.1	46	25.3	0.868 (0.613–1.229)	0.425

* Statistical significance.

**Table 6:** Associations of rs708272 genotypes with MI.

Sex	Genotype	Study Sample		Myocardial Infarction		OR (95% CI)	*p*
		*n*	%	*n*	%		
Males	AA	191	22.4	32	25.0	1.155 (0.751–1.778)	0.511
	AG	389	45.6	47	36.7	0.692 (0.472–1.016)	0.059
	GG	273	32.0	49	38.3	1.138 (0.897–1.935)	0.158
Females	AA	257	20.6	12	21.8	1.078 (0.560–2.075)	0.821
	AG	631	50.5	28	50.9	1.017 (0.593–1.746)	0.950
	GG	362	28.9	15	27.3	0.920 (0.502–1.686)	0.787
Total	AA	448	21.3	44	24.0	1.169 (0.820–1.667)	0.387
	AG	1020	48.5	75	41.0	0.737 (0.543–1.002)	0.051
	GG	635	30.2	64	35.0	1.243 (0.905–1.708)	0.178

**Table 7:** Associations of rs429358 and rs7412 genotypes with MI.

Sex	Genotype	Study Sample		Myocardial Infarction		OR (95% CI)	*p*
		*n*	%	*n*	%		
Males	ɛ2/ɛ4	18	2.1	10	7.8	3.931 (1.772–8.720)	<0.0001 *
	ɛ2/ɛ2	6	0.7	1	0.8	1.112 (0.133–9.309)	0.922
	ɛ2/ɛ3	128	15.0	18	14.0	0.927 (0.544–1.579)	0.780
	ɛ3/ɛ3	525	61.5	81	63.3	1.077 (0.733–1.582)	0.707
	ɛ3/ɛ4	161	18.9	17	13.3	0.658 (0.384–1.128)	0.126
	ɛ4/ɛ4	15	1.8	1	0.8	0.440 (0.058–3.359)	0.416
Females	ɛ2/ɛ4	24	1.9	3	5.5	2.947 (0.860–10.102)	0.071
	ɛ2/ɛ2	11	0.9	-	-	-	-
	ɛ2/ɛ3	155	12.4	6	10.9	0.865 (0.365–2.053)	0.742
	ɛ3/ɛ3	778	62.2	32	58.2	0.844 (0.448–1.460)	0.544
	ɛ3/ɛ4	256	20.5	13	23.6	1.202 (0.635–2.273)	0.571
	ɛ4/ɛ4	26	2.1	1	1.8	0.872 (0.116–6.544)	0.894
Total	ɛ2/ɛ4	42	2.0	13	7.1	3.753 (1.976–7.127)	<0.0001 *
	ɛ2/ɛ2	17	0.8	1	0.6	0.674 (0.089–5.095)	0.701
	ɛ2/ɛ3	283	13.5	24	13.1	0.971 (0.621–1.518)	0.896
	ɛ3/ɛ3	1303	62.0	113	61.7	0.991 (0.726–1.352)	0.955
	ɛ3/ɛ4	417	19.8	30	16.4	0.793 (0.528–1.190)	0.261
	ɛ4/ɛ4	41	1.9	2	1.1	0.556 (0.133–2.316)	0.413

* Statistical significance.

## Data Availability

Raw data are available upon request from the corresponding author. These data are not publicly available due to privacy concerns.
